# Multiresolution Analysis Using Wavelet, Ridgelet, and Curvelet Transforms for Medical Image Segmentation

**DOI:** 10.1155/2011/136034

**Published:** 2011-09-12

**Authors:** Shadi AlZubi, Naveed Islam, Maysam Abbod

**Affiliations:** Department of Electronic and Computer Engineering, School of Engineering and Design, Brunel University, West London UB8 3PH, UK

## Abstract

The experimental study presented in this paper is aimed at the development of an automatic image segmentation system for classifying region of interest (ROI) in medical images which are obtained from different medical scanners such as PET, CT, or MRI. Multiresolution analysis (MRA) using wavelet, ridgelet, and curvelet transforms has been used in the proposed segmentation system. It is particularly a challenging task to classify cancers in human organs in scanners output using shape or gray-level information; organs shape changes throw different slices in medical stack and the gray-level intensity overlap in soft tissues. Curvelet transform is a new extension of wavelet and ridgelet transforms which aims to deal with interesting phenomena occurring along curves. Curvelet transforms has been tested on medical data sets, and results are compared with those obtained from the other transforms. Tests indicate that using curvelet significantly improves the classification of abnormal tissues in the scans and reduce the surrounding noise.

## 1. Introduction

In the last decade, the use of 3D image processing has been increased especially for medical applications; this leads to increase the qualified radiologists' number who navigate, view, analyse, segment, and interpret medical images. The analysis and visualization of the image stack received from the acquisition devices are difficult to evaluate due to the quantity of clinical data and the amount of noise existing in medical images due to the scanners itself. Computerized analysis and automated information systems can offer help dealing with the large amounts of data, and new image processing techniques may help to denoise those images.

Multiresolution analysis (MRA) [1–3] has been successfully used in image processing specially with image segmentation, wavelet-based features has been used in various applications including image compression [4], denoising [5], and classification [6]. Recently, the finite ridgelet and curvelet transforms have been introduced as a higher dimensional MRA tool [7, 8].

Image segmentation requires extracting specific features from an image by distinguishing objects from the background. The process involves classifying each pixel of an image into a set of distinct classes, where the number of classes is much smaller. Medical image segmentation aims to separate known anatomical structures from the background such cancer diagnosis, quantification of tissue volumes, radiotherapy treatment planning, and study of anatomical structures.

Segmentation can be manually performed by a human expert who simply examines an image, determines borders between regions, and classifies each region. This is perhaps the most reliable and accurate method of image segmentation, because the human visual system is immensely complex and well suited to the task. But the limitation starts in volumetric images due to the quantity of clinical data.

Curvelet transform is a new extension of wavelet transform which aims to deal with interesting phenomena occurring along curved edges in 2D images [9]. It is a high-dimensional generalization of the wavelet transform designed to represent images at different scales and different orientations (angles). It is viewed as a multiscale pyramid with frame elements indexed by location, scale, and orientation parameters with needle-shaped elements at fine scales. Curvelets have time-frequency localization properties of wavelets but also shows a very high degree of directionality and anisotropy, and its singularities can be well approximated with very few coefficients. 

This paper is focusing on a robust implementation of MRA techniques for segmenting medical volumes using features derived from the wavelet, ridgelet, and curvelet transforms of medical images obtained from a CT scanner. The rest of this paper is organised as follow: Section 2 illustrates the proposed medical image segmentation system using MRA techniques. The mathematical background and the methodology for the proposed MRA techniques have been explained in Section 3. The results and analysis of the implemented wavelet, ridgelet, and curvelet transforms for medical image segmentation are illustrated in Section 4. Finally, Section 5 includes the conclusions and future work of this research. 

## 2. Proposed Medical Image Segmentation System

**Figure 1:**
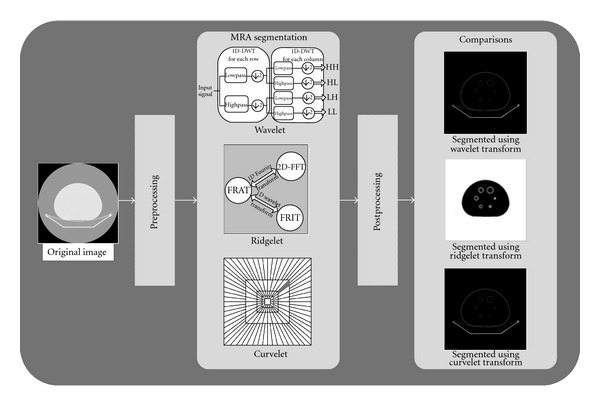
Proposed segmentation system for medical images.

The main aim of this research is to facilitate the process of highlighting ROI in medical images, which may be encapsulated within other objects or surrounded by noise that make the segmentation process not easy.  Figure 1 illustrates the proposed medical image segmentation system using MRA. Wavelet, ridgelet, and curvelet transforms are applied on medical images with other pre- and postprocessing techniques to present segmented outputs and detected ROI in an easier and more accurate way.

## 3. Methodology—Multiresolution Analysis

Image segmentation using MRA such as wavelets has been widely used in recent years and provides better accuracy in segmenting different types of images. Many recent developments in MRA have taken place, while wavelets are suitable for dealing with objects with point singularities. Wavelets can only capture limited directional information due to its poor orientation selectivity. By decomposing the image into a series of high-pass and low-pass filter bands, the wavelet transform extracts directional details that capture horizontal, vertical, and diagonal activity. However, these three linear directions are limiting and might not capture enough directional information in noisy images, such as medical CT scans, which do not have strong horizontal, vertical, or diagonal directional elements. Ridgelet improves MRA segmentation; however, they capture structural information of an image based on multiple radial directions in the frequency domain. Line singularities in ridgelet transform provides better edge detection than its wavelet counterpart. One limitation to use ridgelet in image segmentation is that ridgelet is most effective in detecting linear radial structures, which are not dominant in medical images. The curvelet transform is a recent extension of ridgelet transform that overcome ridgelet weaknesses in medical image segmentation. Curvelet is proven to be particularly effective at detecting image activity along curves instead of radial directions which are the most comprising objects of medical images. 

### 3.1. Wavelet Transform

In the last decade, wavelet transform has been recognized as a powerful tool in a wide range of applications, including image/video processing, numerical analysis, and telecommunication. The advantage of wavelet is that wavelet performs an MRA of a signal with localization in both time and frequency [10, 11]. In addition to this, functions with discontinuities and functions with sharp spikes require fewer wavelet basis vectors in the wavelet domain than sine cosine basis vectors to achieve a comparable approximation. Wavelet operates by convolving the target function with wavelet kernels to obtain wavelet coefficients representing the contributions in the function at different scales and orientations. Wavelet or multiresolution theory can be used alongside segmentation approaches, creating new systems which can provide a segmentation of superior quality to those segmentation approaches computed exclusively within the spatial domain [12].

Discrete wavelet transform (DWT) can be implemented as a set of high-pass and low-pass filter banks. In standard wavelet decomposition, the output from the low-pass filter can be then decomposed further, with the process continuing recursively in this manner. According to [13], DWT can be mathematically expressed by

(1) $$\begin{array}{cc} a^{j}\left(n\right) & =\overset{L-1}{\underset{i=0}{\sum }}l\left(i\right)\cdot a^{j-1}\left(2n-i\right),\;0\leq n<N_{j},\\ d^{j}\left(n\right) & =\overset{L-1}{\underset{i=0}{\sum }}h\left(i\right)\cdot d^{j-1}\left(2n-i\right),\;0\leq n<N_{j}. \end{array}$$

The coefficients *a*^*j*^(*n*) and *d*^*j*^(*n*) refer to approximation and detailed components in the signal at decomposition level *j*, respectively. The *l*(*i*) and *h*(*i*) represent the coefficients of low-pass and high-pass filters, respectively.

DWT decomposes the signal into a set of resolution-related views. The wavelet decomposition of an image creates at each scale *j* a set of coefficient values *w*_*j*_ with an overall mean of zero. *w*_*j*_ contains the same number of voxels as the original image; therefore, this wavelet transform is redundant [14, 15]. 

**Figure 2:**
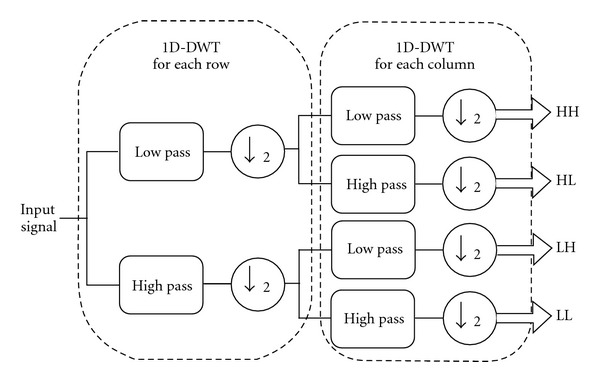
2D DWT filter structure.

For images, 1D-DWT can be readily extended into 2D. In standard 2D wavelet decomposition, the image rows are fully decomposed, with the output being fully decomposed columnwise. In nonstandard wavelet decomposition, all the rows are decomposed by one decomposition level followed by one decomposition level of the columns.  Figure 2 illustrates the filter structure of 2D-DWT.

**Figure 3:**
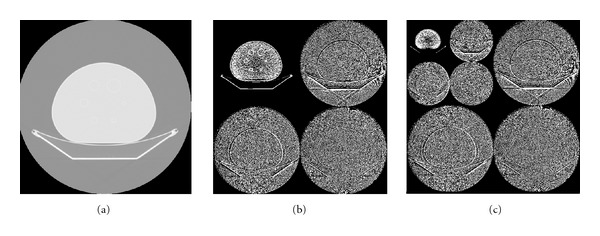
2D-DWT. Original image (a), first decomposition level (b), and second decomposition level (c).

Wavelet uses a set of filters to decompose images depending on filter coefficients and the number of those coefficients. The most popular wavelet filter is Haar wavelet filter (HWF) which takes the averages and differences from the low- and high-pass filters, respectively.  Figure 3 illustrates an example of applying 2D-DWT using HWF on an image for 2 levels of decompositions.

### 3.2. Ridgelet Transform

In 1998, Donoho introduced the ridgelet transform [16] continuous ridgelet transform (CRT) can be defined from a 1D wavelet function oriented at constant lines and radial directions. Ridgelet transform [17–19] has been generating a lot of interest due to their superior performance over wavelets. While wavelets have been very successful in applications such as denoising and compact approximations of images containing zero dimensional or point singularities. Wavelets do not isolate the smoothness along edges that occurs in images [20], and they are thus more appropriate for the reconstruction of sharp point singularities than lines or edges. These shortcomings of wavelet are well addressed by the ridgelet transform; the functionality of wavelet has been extended to higher dimensional singularities and becomes an effective tool to perform sparse directional analysis [3, 21]. Generally speaking, wavelets detect objects with point singularities, while ridgelets are able to represent objects with line singularities. 

The finite ridgelet transform (FRIT) was computed in two steps: a calculation of discrete radon transform and an application of a wavelet transform. The finite radon transform (FRAT) is computed in two steps: a calculation of 2D Fast Fourier Transform (FFT) for the image and an application of a 1D inverse fast Fourier transform (iFFT) on each of the 32 radial directions of the radon projection. 1D wavelet is applied restricted to radial directions going through the origin for three levels of decompositions.

Applying FRAT on image can be presented as a set of projections of the image taken at different angles to map the image space to projection space. Its computation is important in image processing and computer vision for problems such as pattern recognition and the reconstruction of medical images. For discrete images, a projection is computed by summation of all data points that lie within specified unit-width strips; those lines are defined in a finite geometry [22].

Depending on [23], FRAT of a real function on the finite grid *Z*_*p*_^2^ is defined in

(2) $$r_{k}\left[l\right]={\text{FRAT}}_{f}\left(k,l\right)=\frac{1}{\sqrt{P}}\underset{\left(i,j\right)\in L\left(k,l\right)}{\sum }f\left(i,j\right).$$

Here, *L*(*k*, *l*) denotes the set of points that make up a line on the lattice *Z*_*p*_^2^ as in

(3) $$\begin{array}{c} L\left(k,l\right)=\left\{\left(i,j\right):j=k_{i}+l\left(\mathrm{mod}\;p\right),i\in Z_{p}\right\},\;0\leq k<p,\\ L\left(p,l\right)=\left\{\left(l,j\right):j\in Z_{p}\right\}. \end{array}\;$$

To compute the *K*th radon projection (i.e., the *K*th row in the array), all pixels of the original image need to be passed once and use *P* histogrammers: one for every pixel in the row [12]. At the end, all *P* histogrammed values are divided by *K* to get the average values.

**Figure 4:**
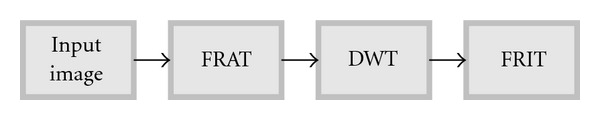
FRIT block diagram.

According to Alzu'bi and Amira in [3], once the wavelet and radon transforms have been implemented, the ridgelet transform is straightforward. Each output of the radon projection is simply passed through the wavelet transform before it reaches the output multiplier. As shown in Figure 4, ridgelets use FRAT as a basic building block, where FRAT maps a line singularity into point singularity, and the wavelet transform has used to effectively detect and segment the point singularity in radon domain.

**Figure 5:**
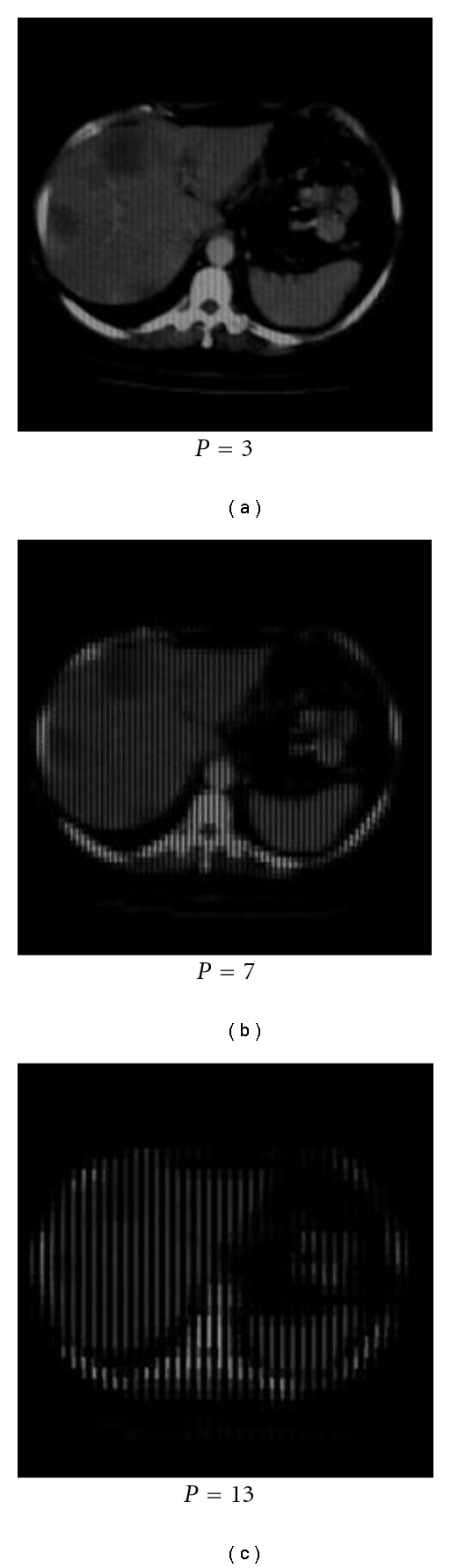
Ridgelet transform for real CT images at block sizes (3, 7, and 13).

Figure 5 shows a clinical chest slice from a CT scanner [24] in the last step of ridgelet transform before image reconstruction at different block sizes.

**Figure 6:**
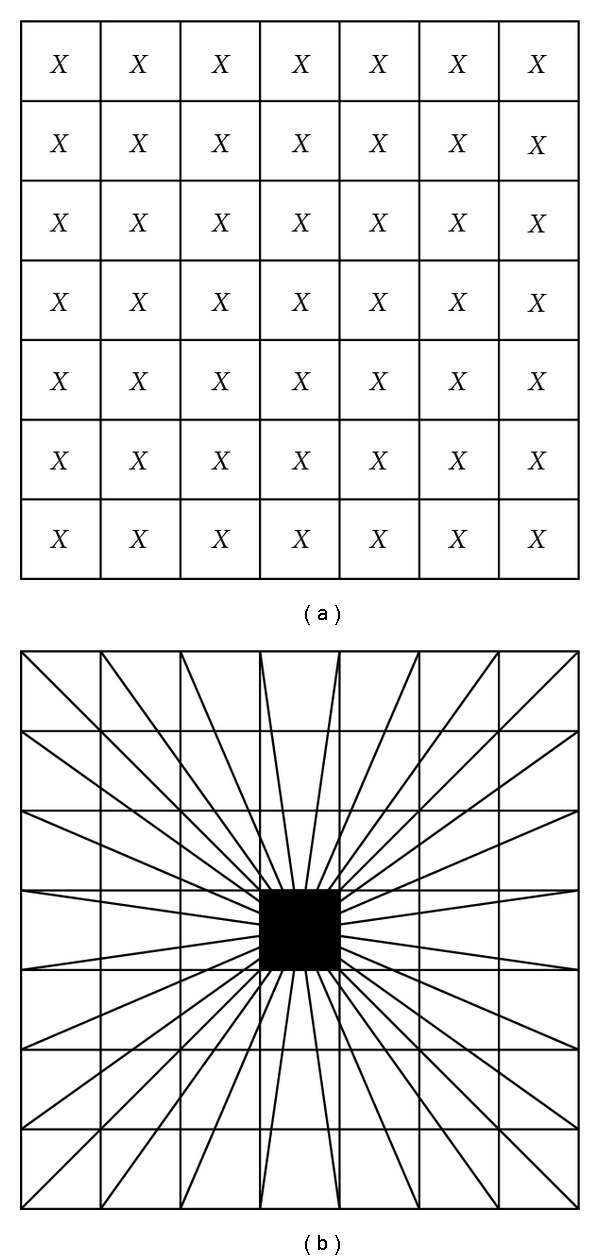
Wavelet and ridgelet parameters.

Continuous ridgelet transform is similar to the continuous wavelet transform except that point parameters (*x*, *y*) in the cartesian grid (Figure 6(a)) which perform pixels in the image or an entry in a 2D matrix are now replaced by line parameters (*β*, *θ*), where *β* is the intercept and *θ* is the angle.  Figure 6(b) illustrates the radial grid in ridgelet transform; however, straight lines evaluate the image in the frequency domain [3].

The segmentation result achieved using ridgelet transformation on medical images was not promising. Medical images comprised from curves which are still not singularity points after applying radon transform. Wavelet transform cannot detect those singularities properly, since it still not singularity points [3], resulting that ridgelet transformation is not suitable for segmenting these images.

Ridgelet transform can be used in other applications, where images contain edges and straight lines. Curvelet transform has been introduced to solve this problem; it deals with higher singularities compared to wavelet and ridgelet transforms.

### 3.3. Curvelet Transform

The curvelet transform has gone through two major revisions. It was first introduced in [25, 26] by Candés and Donoho in 2000, which used a complex series of steps involving the ridgelet analysis of the radon transform of an image. Their performance was very slow; hence, researchers developed a new version which is easier to use and understand. In this new method, the use of the ridgelet transform as a preprocessing step of curvelet was discarded, thus reducing the amount of redundancy in the transform and increasing the speed considerably [3].

**Figure 7:**
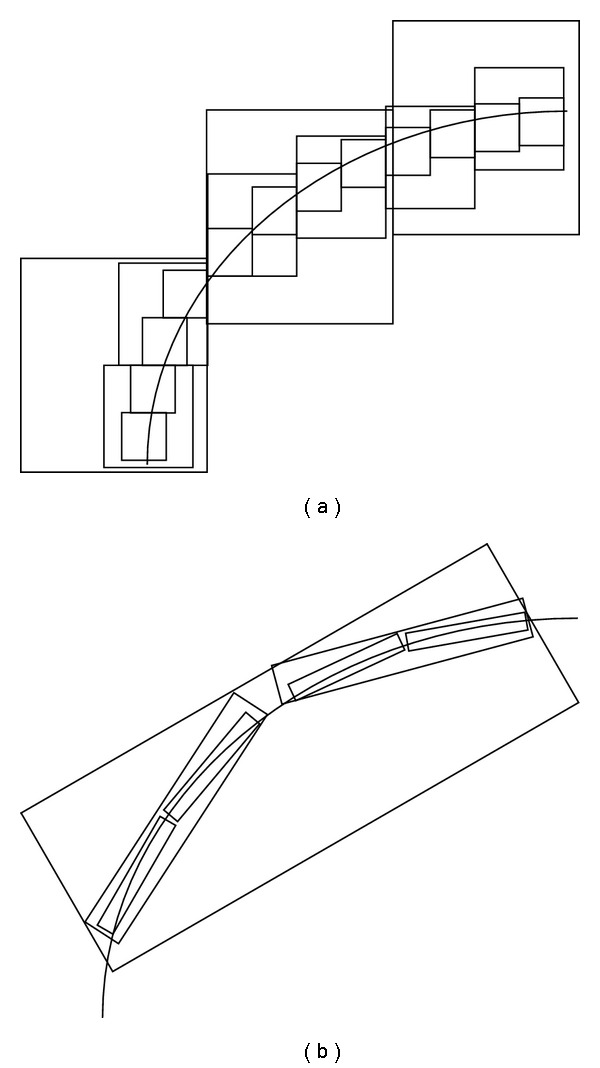
An approximating comparison between wavelet (a) and curvelet (b).

Curvelet aims to deal with interesting phenomena occurring along curved edges in a 2D image. As illustrated in Figure 7, curvelet needs fewer coefficients for representation, and the edge produced from curvelet is smoother than wavelet edge [27].

The newly constructed and improved version of curvelet transform is known as Fast Discrete Curvelet Transform (FDCT). This new technique is simpler, faster and less redundant than the original curvelet transform which based on ridgelets. According to Candes et al. in [9], two implementations of FDCT are proposed:

- unequally spaced Fast Fourier transforms (USFFT),
- wrapping function.

Both implementations of FDCT differ mainly by the choice of spatial grid that used to translate curvelets at each scale and angle. Both digital transformations return a table of digital curvelet coefficients indexed by a scale parameter, an orientation parameter, and a spatial location parameter. Wrapping-based transform is based on wrapping a specially selected Fourier samples, and it is easier to implement and understand.

#### 3.3.1. Continuous-Time Curvelet Transform

Curvelet transform works in two dimensions with spatial variable *x*, frequency domain variable *ω*, and the frequency-domain polar coordinates *r* and *θ*. Curvelet transform can be defined by a pair of windows, radial window {*W*(*r*)}, and angular window {*V*(*t*)} [9]. As illustrated in (4), these windows will always obey the admissibility conditions.

(4) $$\begin{array}{cc} \overset{\infty }{\underset{j=-\infty }{\sum }}W^{2}\left(2^{j}r\right) & =1,\;r\in \left(\frac{3}{4},\frac{3}{2}\right),\\ \overset{\infty }{\underset{j=-\infty }{\sum }}V^{2}\left(t-l\right) & =1,\;t\in \left(-\frac{1}{2},\;\frac{1}{2}\right). \end{array}$$

A polar “wedge” represented by *U*_*j*_ is supported by the radial window {*W*(*r*)} and angular window {*V*(*r*)}. Equation (5) defines *U*_*j*_ in the Fourier domain

(5) $$\begin{array}{cc} U_{j}\left(r,\theta \right) & =2^{-3j/4}W\left(2^{-j}r\right)V\left(\frac{2^{\left\lfloor j/2\right\rfloor }\theta }{2\pi }\right). \end{array}$$

**Figure 8:**
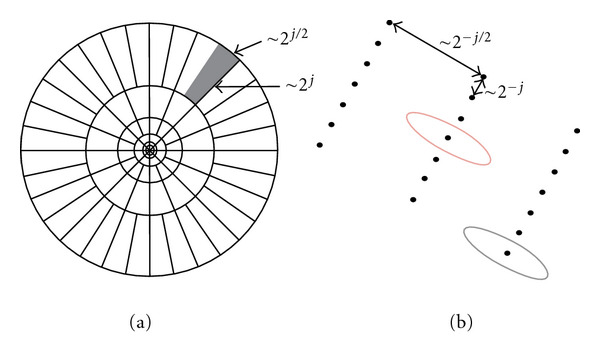
Curvelet tiling of space and frequency. The induced tiling of the frequency plane (a). The spatial Cartesian grid associated with a given scale and orientation (b).

Equation (6) defines the curvelet transform as a function of {*x* = (*x*_1_, *x*_2_)} at scale 2^−*j*^, orientation *θ*_*l*_ and position *x*_*k*_(*j*, *l*), where *R*_*θ*_ is the rotation in radians.  Figure 8 illustrates the induced tiling of the frequency plane and the spatial Cartesian grid associated with a given scale and orientation [9], and shaded area represents the polar wedge by *U*_*j*_

(6) $$\varphi_{j,l,k}\left(x\right)=\varphi_{j}\left(R_{\theta_{l}}\left(x-x_{k}^{\left(j,l\right)}\right)\right).$$

#### 3.3.2. Fast Discrete Curvelet Transform via Wrapping

The new implementation of curvelet transform based on Wrapping of Fourier samples takes a 2D image as an input in the form of a Cartesian array *f*[*m*, *n*], where 0 ≤ *m* < *M*, 0 ≤ *n* < *N* where *M* and *N* are the dimensions of the array. As illustrated in (7), the outputs will be a collection of curvelet coefficients *c*^*D*^(*j*, *l*, *k*_1_*k*_2_)  indexed by a scale *j*, an orientation *l* and spatial location parameters *k*_1_ and *k*_2_.

(7) $$c^{D}\left(j,l,k_{1}k_{2}\right)=\overset{0\leq m<M}{\underset{0\leq n<N}{\sum }}f\left[m,n\right]\varphi_{j,l,k_{1}k_{2}}^{D}\left[m,n\right].$$

Each *φ*_*j*,*l*,*k*_1_*k*_2__^*D*^ is a digital curvelet waveform, superscript *D* stands for “digital.” These approach implementations are the effective parabolic scaling law on the subbands in the frequency domain to capture curved edges within an image in more effective way. As mentioned earlier, wrapping based curvelet transform is a multiscale pyramid which consists of several subbands at different scales consisting of different orientations and positions in the frequency domain. At a high frequency level, curvelets are so fine and looks like a needle shaped element and they are non-directional coarse elements at low frequency level. 

**Figure 9:**
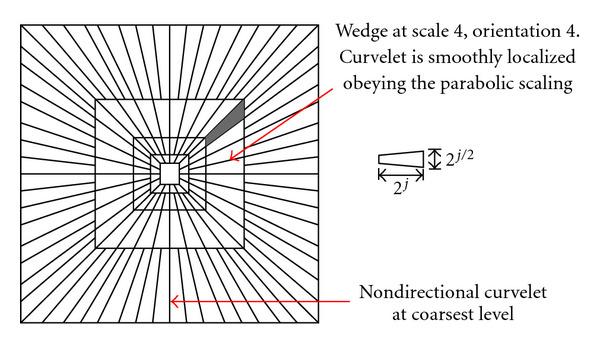
5-level curvelet digital tiling of an image.

Figure 9 illustrates the whole image represented in spectral domain in the form of rectangular frequency tiling by combining all frequency responses of curvelets at different scales and orientations. It can be seen that curvelets are needle like elements at higher scale.

It can be seen from Figure 9 that curvelet becomes finer and smaller in the spatial domain and shows more sensitivity to curved edges as the resolution level is increased, thus allowing to effectively capturing the curves in an image, and curved singularities can be well-approximated with fewer coefficients. 

In order to achieve a higher level of efficiency, curvelet transform is usually implemented in the frequency domain. This means that a 2D FFT is applied to the image. For each scale and orientation, a product of *U*_*jl*_ “wedge” is obtained; the result is then wrapped around the origin, and 2D IFFT is then applied resulting in discrete curvelet coefficients. Candes et al. describe the discrete curvelet transform in [9] as illustrated in

(8) Curvelet  transform=IFFT[FFT(Curvelet)×FFT(Image)].

**Figure 10:**
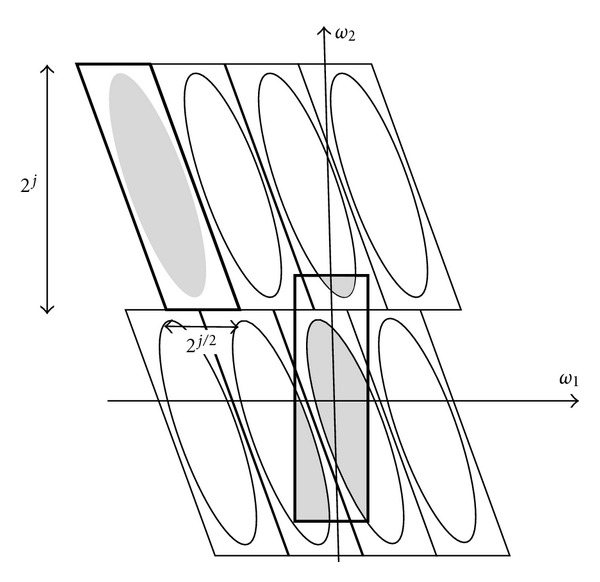
Wrapping wedge data.

The difficulty behind this is that trapezoidal wedge does not fit in a rectangle of size 2^*j*^ × 2^*j*/2^ aligned with the axes in the frequency plane in which the 2D IFFT could be applied to collect curvelet coefficients. Wedge wrapping procedure proposed in [9] uses a parallelogram with sides 2^*j*^ and 2^*j*/2^ to support the wedge data. The wrapping is done by periodic tiling of the spectrum inside the wedge and then collecting the rectangular coefficient area in the centre. The centre rectangle of size 2^*j*^ × 2^*j*/2^ successfully collects all the information in that parallelogram [28].  Figure 10 illustrates the process of wrapping wedge where the angle *θ* is in the range (*π*/4, 3*π*/4) and the rectangles have the same width and length as the parallelogram is centred at the origin [9].

The following are the steps of applying wrapping based FDCT algorithm [9].

**Step 1**

Apply the 2D FFT to an image to obtain Fourier samples

(9) $$\hat{f}\left[m,n\right],\;-\frac{n}{2}\leq m,\;n<\frac{n}{2}.$$

**Step 2**

For each scale *j* and angle *l*, form the product

(10) $${\tilde{U}}_{j,l}\left[m,n\right]\hat{f}\left[m,n\right].$$

**Step 3**

Wrap this product around the origin and obtain

(11) $${\tilde{f}}_{j,l}\left[m,n\right]=W\left({\tilde{U}}_{j,l}\hat{f}\right)\left[m,n\right],$$

where the range for *m*, *n*, and *θ* is now 0 ≤ *m* < 2^*j*^, 0 ≤ *n* < 2^*j*/2^, and −*π*/4 ≤ *θ* < *π*/4.

**Step 4**

Apply IFFT to each$\;{\tilde{f}}_{j,l}$, hence collecting the discrete coefficients *c*^*D*^(*j*, *l*, *k*_1_*k*_2_).

The curvelet transform is a multiscale transform such as wavelet, with frame elements indexed by scale and location parameters. Wavelets are only suitable for objects with point singularities, Ridgelets are only suitable for objects with line singularities, while curvelets have directional parameters and its pyramid contains elements with a very high degree of directional specificity. The elements obey a special scaling law, where the length and the width of frame elements support are linked using

(12) $$\text{width}\approx {\text{length}}^{\text{2}}$$

Discrete curvelet transform in the spectral domain utilizes the advantages of FFT. During FFT, both image and curvelet at a given scale and orientation are transformed into the Fourier domain. The convolution of the curvelet with the image in the spatial domain then becomes their product in the Fourier domain. At the end of this computation process, a set of curvelet coefficients are obtained by applying IFFT to the spectral product. This set contains curvelet coefficients in ascending order of the scales and orientations. 

Curvelets are superior to the other transforms as in the following.

**(a) Optimally Sparse Representation of Objects with Edges**

Curvelets provide optimally sparse representation of objects which display curve-punctuated smoothness except for discontinuity along a general curve with a bounded curvature. Such representations are nearly as sparse as if the object were not singular and turn out to be far sparser than other transforms decomposition of the object.

**(b) Optimal Image Reconstruction in Severely Ill-Posed Problems**

Curvelets also have special microlocal features which make them especially adapted to certain reconstruction problems with missing data. For example, in many important medical applications, one wishes to reconstruct an object *f*(*x*_1_, *x*_2_) from noisy and incomplete tomographic data [28]. Because of its relevance in biomedical imaging, this problem has been extensively studied, yet curvelets offer surprisingly new quantitative insights [18]. For example, an application of the phase-space localization of the curvelet transform allows a very precise description of those features of the object of function (*f*) which can be reconstructed accurately from such data and how well, and of those features which cannot be recovered.

As illustrated in (8), the data acquisition geometry separates the curvelet expansion of the object into two pieces as illustrated in

(13) $$f=\underset{n\in \text{Good}}{\sum }\langle f,\varphi_{n}\rangle \varphi_{n}+\underset{n\notin \text{Good}}{\sum }\langle f,\varphi_{n}\rangle \varphi_{n}.$$

The first part of (13) can be recovered accurately, while the second part cannot. What is interesting here is that one can provably reconstruct the recoverable part with an accuracy similar to that one would achieve even if one had complete data. There is indeed a quantitative theory showing that for some statistical models which allow for discontinuities in the object to be recovered, there are simple algorithms based on the shrinkage of curvelet biorthogonal decompositions, which achieve optimal statistical rates of convergence [18].

**Figure 11:**
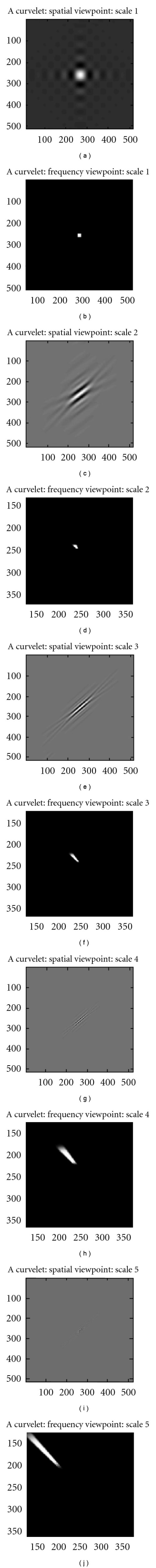
Curvelets at increasingly fine scales from 1 to 5. Spatial domain (a, c, e, g, i). Frequency domain (b, d, f, h, j) [29].

Figure 11 illustrates the frequency response of curvelets at different scales and orientations for some test images using “*Curvelab (version: 2.1.2)*” [29] in both spatial and frequency domain.

**Figure 12:**
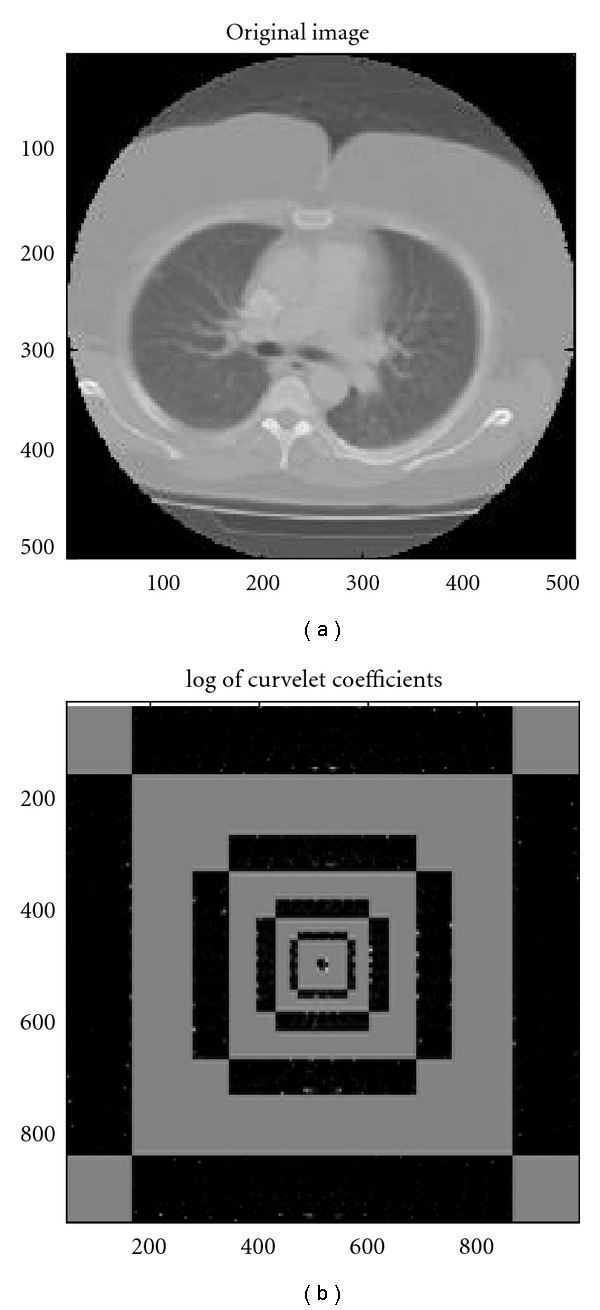
Clinical slice for the human chest from a CT scanner in spatial (a) and curvelet coefficients (b).

It can be seen from Figure 11 that curvelets are nondirectional at coarsest level. Figures 11(a), 11(c), 11(e), 11(g), and 11(i) are the spatial representation of curvelet at scales 1 to 5. And Figures 11(b), 11(d), 11(f), 11(h), and 11(j) are the frequency domain representation of curvelet that is modulus of FFT.  Figure 12 illustrates a clinical data for human chest from CT scanner in spatial domain and its curvelet coefficients.

In Figure 12, the low frequency coefficients (coarse scale) are stored at the centre of the display. The concentric coronae (formed by black strips) show coefficients at different scales and the outer coronae correspond to higher frequencies. Each corona has four strips further subdivided in angular panels; each panel represents coefficients at a specified scale and orientation suggested by the position of the panel. 

Wedge wrapping is done for all the wedges at each scale in the frequency domain to obtain a set of subbands or wedges at each curvelet decomposition level, and these subbands are the collection of discrete curvelet coefficients. 

**Figure 13:**
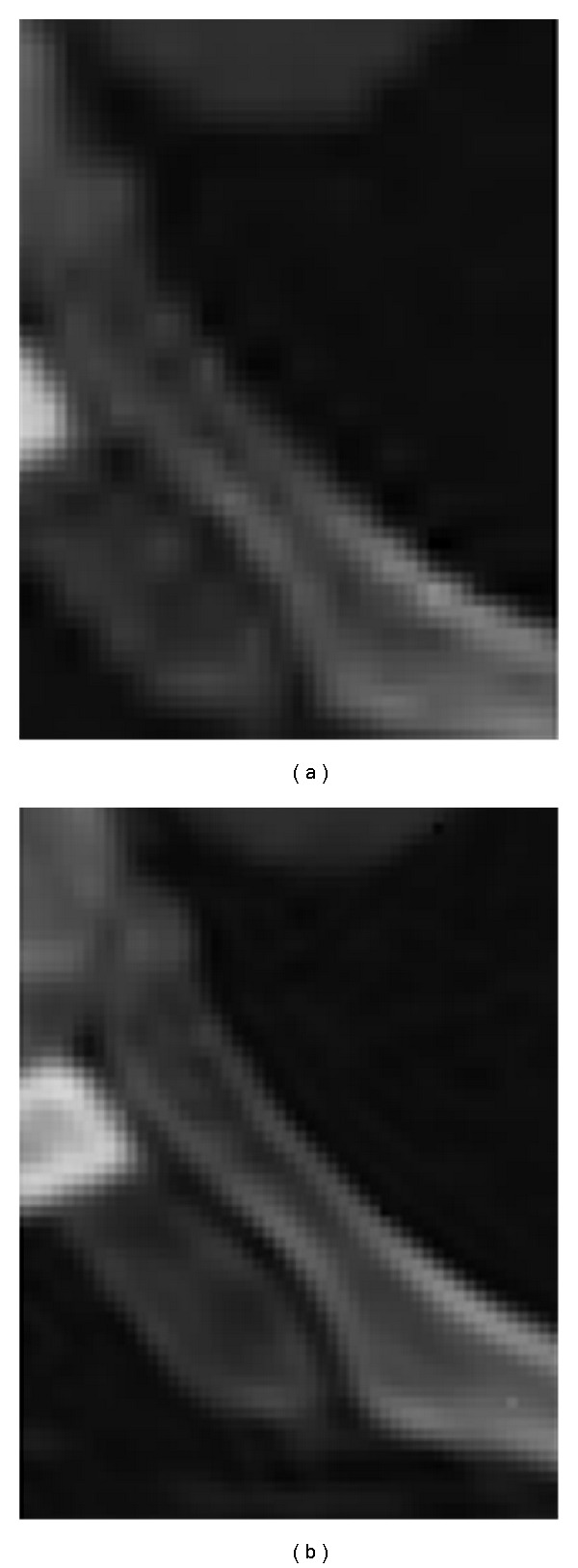
Reconstruction of tomographic data. Wavelet domain (a), and curvelet domain (b).

The aim is to identify the most effective texture descriptor for medical images to capture edge information more accurately. The discrete curvelet transform can be calculated to various resolutions or scales and angles; the maximum number of resolution depends on the original image size and the angles. Number of angles at the second coarsest level must be at least eight and a multiple of four; that is, 512 × 512 image has five maximum possible resolution levels containing structural information of the image. Figure 13 illustrates how curvelet-based edge reconstruction in medical imaging differs from other transforms methods. 

## 4. Results and Analysis

The end users of the proposed system are the radiologists and specialists who analyse medical images for cancer diagnosis. After several meetings with those people in the radiology departments in some hospitals, the main goal that they are working is to detect the accurate cancer size in medical images with the least error. This process may be affected by the noise surrounding ROI, which make the process of measuring the exact dimensions of the lesion so hard.

Different datasets have been carried out with the proposed system to validate it for clinical applications. The first one is NEMA IEC body phantom which consists of an elliptical water filled cavity with six spherical inserts suspended by plastic rods of inner diameters: 10, 13, 17, 22, 28, and 37 mm [25, 26]. Real clinical human images acquired by a CT scanner [24] have also been used to experiment the proposed approaches, this data has been previously analysed by the radiologists and the provided reports explains that the patients are diagnosed by cancer.  Table 1 illustrates the SNR values of extracted features from NEMA IEC DATA SET in spatial domain, different levels of decomposition of wavelet domain and at different block sizes in ridgelet domain.

It can be seen from Table 1 that small values of SNR have been obtained for all techniques; this is due to the noise from the acquisition systems itself. This noise will be a part of the medical image after the reconstruction of all slices. Relatively, better SNR values can be achieved with the second level of wavelet decomposition and as the block size (*p*) is getting bigger with the ridgelet transform, where the transformed image is getting more similar to the original image. This can be assigned to the major limitation of using ridgelet transformation in medical image segmentation, where ridges rarely exist in such data.

MRA transforms have been used with thresholding technique to segment the experimental data. Thresholding technique has been applied as a preprocessing step on the original images at threshold value (*t* = 35) to remove as much artificial spam sequel produced from the scanners. The transform then applied to effectively represent objects with edges which are the contours of the medical images followed by another thresholding at (*t* = 7) to remove most of the remaining noise and facilitate the measurement process.

**Figure 14:**
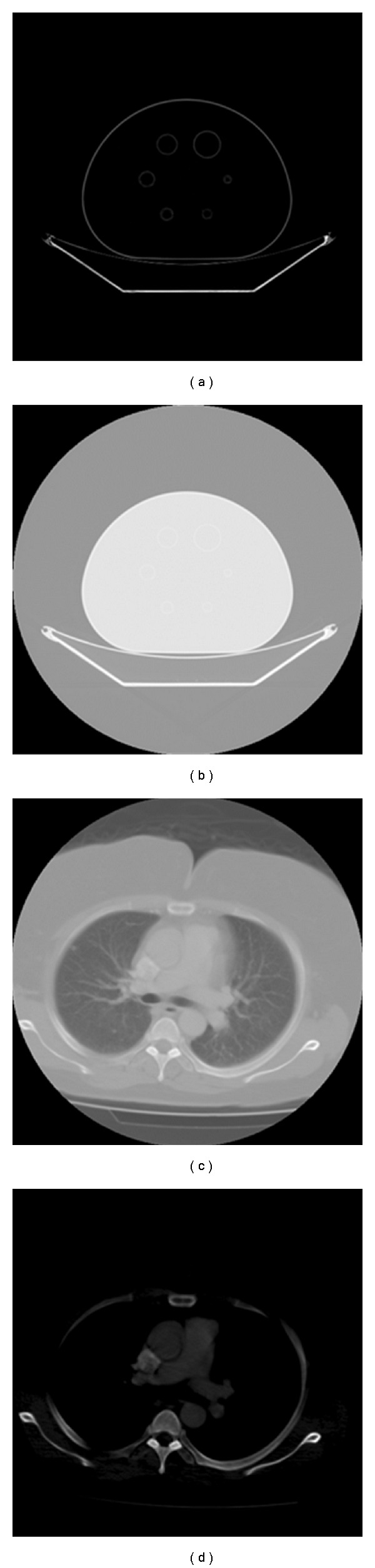
Curvelet transform for segmentation. (a) NEMA IEC body phantom, (b) segmented phantom slice, (c) original real chest slice, and (d) segmented real chest slice.

**Figure 15:**
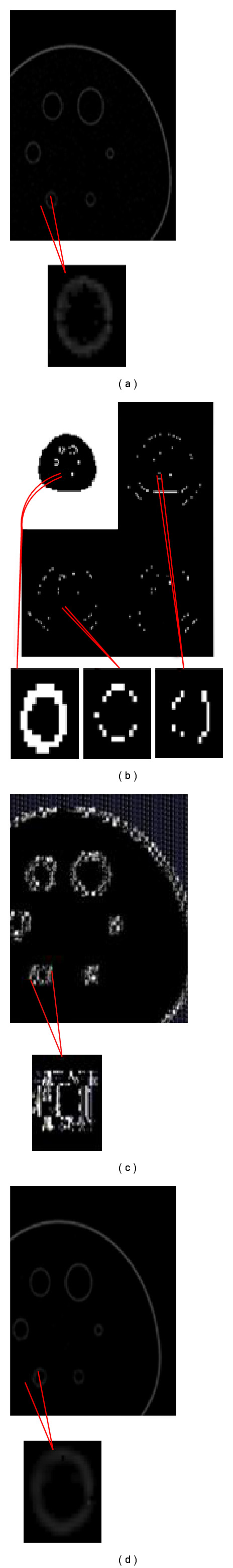
Segmentation using conventional hard thresholding and curvelet-based segmentation. (a) Denoised spatial domain. (b) First level in wavelet domain. (c) Ridgelet domain. (d) Curvelet domain.

Figure 14 illustrates the segmentation using curvelet transform. Figures 14(a) and 14(c) illustrate the original images from a CT scanner, and Figures 14(b) and 14(d) illustrate the segmented phantom image and real chest image, respectively, using curvelet transform. As illustrated in Figure 15, results of the proposed segmentation technique are vary in terms of smooth reconstruction of the spheres. Curvelet transform segments the input image and removes artifacts from the image to exhibit smooth and optimal segmentation of NEMA phantom. Ridgelet transform detect ROI but does not give promising segmentation results due to the lack of ridges or straight lines in the tested data set. Wavelet quadrants are varying also in their quality; relatively, the best results have been achieved with the LL-filter output.

Table 2 illustrates NEMA spheres diameters error percentages measured using different multiresolution analysis techniques and compared to previously implemented techniques. ED has been used to measure the spheres diameters and calculate the error percentages for each technique, and sphere diameter error percentages have been calculated as follows:

(14) $$\text{error}\;\%=\frac{\text{Measured}\;\text{Diameter}-\text{Actual}\;\text{Diameter}}{\text{Actual}\;\text{Diameter}}\times 100\%.$$

From Table 2, in the case of K-means clustering, tumor volumes are underestimated by approximately 5%-6% in most cases; however, for the two smaller spherical inserts, with diameter of 10 mm and 13 mm, respectively, these underestimations are significantly greater. For the smallest sphere, more than a 13% volume discrepancy is recorded, with the K-means algorithm finding it difficult to quantify the tumor accurately. Sphere 2 similarly is massively underestimated (11.5%). Unlike K-means clustering, MRFM tends to overestimate the volumes of the spherical inserts, with the exception of Spheres 1 and 2.

Spheres diameters are reduced to the half with each decomposition level of wavelet transform. Three decomposition levels of DWT have been applied on NEMA phantom [25, 26] using two different filters (Haar, Daubechies), and the measured diameters were doubled at each level to produce a fair comparison with the other available techniques. It can be seen that most of the error percentages were decreasing while the spheres diameter increasing; it is worth mentioning that there is no upper bound of the spheres diameters to keep the errors decreasing, because the ROI becomes clearer and easier to be detected and measured properly. But tumours in real life are usually very small in the early stage cancer, and the problem is to detect those turnouts' tumours as soon as possible.

**Figure 16:**
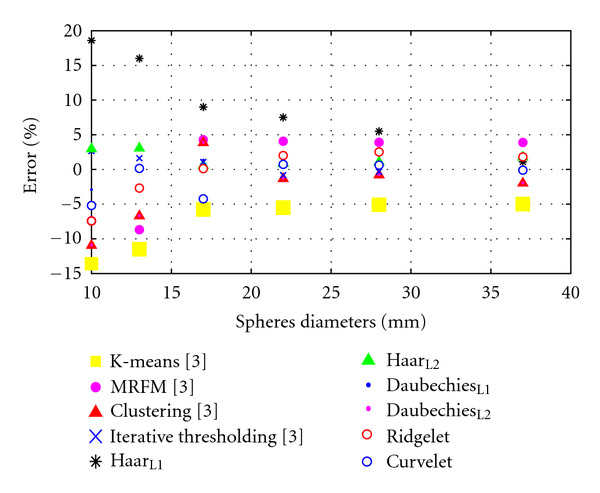
Visual comparison for error percentages in Table 2.

The two smallest spherical inserts are still underestimated in most of the techniques and got the largest error percentages. The large volumetric errors encountered using this acquisition exist as a consequence of the poor slice thickness setting selected for the scan. The 4.25 mm slice thickness causes large fluctuations in transaxial tumour areas to occur between image slices. This problematic characteristic occurs most notably with the smallest spherical inserts, where single voxel reallocation causes a large deviation in percentage error. In Figure 16, the percentage error computed between the actual sphere volume and the volumes obtained using all methodologies for each of the six tumours inserts is plotted. It can be seen that all techniques are settled down according to the error percentages as the sphere diameters increased.

It can be also seen from Table 2 that acceptable error percentages have been achieved using ridgelet transform for the big spheres (22 mm, 28 mm, and 37 mm), where the curves are not sharp and ridgelet detect it accurately as it close to be ridges. But for the small spheres, ridgelet weakness for medical image segmentation start appears clearly.

Curvelet transform overcomes the weakness of wavelet for segmenting sharp curves and detect the small spheres accurately with error percentages (0.82%–2.65%). For the big spheres, errors achieved using wavelet transform are still better than those achieved using curvelet transform due to the sharpness of that spheres. But still very good results using curvelet transform and acceptable for clinical applications.

PSNR and MSE have been also used to evaluate the quality of the proposed techniques. The original image has been contaminated with Gaussian white noise at *σ* = 20% of the maximum intensity.  Table 3 illustrates a comparison study of curvelet transform with the other traditional transforms, and comparison terms PSNR and MSE have been used to test the quality of the transformed image.

**Figure 17:**
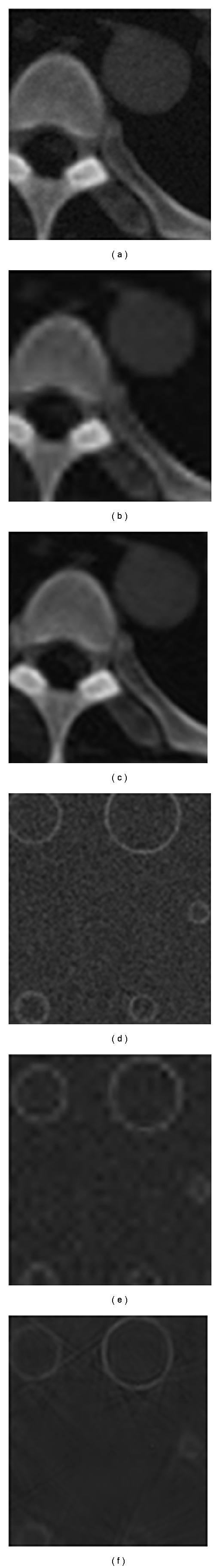
MRA for image denoising. (a, d) Noisy images. (b, e) Wavelet. (c, f) Curvelet.

From Table 3, it can be seen that the best results according to both PSNR and MSE have been achieved using curvelet transform. Wavelet transform performs better results compared to ridgelet transform in both validation metrics.  Figure 17 illustrates two noisy images and the denoised outputs using both wavelet and curvelet.

According to a study done by Dettori and Semler [23], the ridgelet-based descriptors had significantly higher performance measures in comparison to wavelet-based descriptors, with accuracy rates higher than any other wavelet-based feature set for all individual organs. This is not surprising given the fact that the ridgelet transform is able to capture multidirectional features, as opposed to the wavelet transform which focus mainly on horizontal, vertical, and diagonal features. This can be generalized to most of the images except for medical scanners, where the weakness of wavelet is not dominant in such images.

Curvelet-based descriptors had an even higher performance in comparison to both the wavelet and ridgelet, with accuracy rates higher, respectively. The accuracy rate using curvelet transform is better; this is expected, since the curvelet transform is able to capture multidirectional features in wedges, as opposed to lines or points as in the ridgelet or wavelet transform. The multidirectional features in curvelets are very effective in extracting the important features from medical images and then segmented accurately.

As illustrated in the previous tables and figures, it can be seen that more efficient and smooth image reconstruction is achieved using curvelet transform. In terms of optimal reconstruction of the objects with edges and curves, curvelet-based techniques outperform the traditional wavelet and ridgelet transforms.

The algorithm presented in this chapter is able to classify normal tissues in CT scans with high accuracy rates. These hypotheses will be further tested and validated on different predefined clinical data sets in chapter 8 of this thesis.

Segmentation using curvelet transform has been chosen for experimenting the PET scanner sensitivity variables, curvelet was applied in parallel with multithresholding and classification techniques to classify the spheres in a separate class from the other comprising objects at least noise included. The experiment was evaluated based on the ratio between the spheres area to the other area of the scanned slice. The actual spheres area can be calculated according to (15), given that the spheres diameters are 10, 13, 17, 22, 28, 37 mm

(15) $$S_{\text{Original}}=\frac{1}{2}\pi \underset{r\in \{a\}}{\sum }r^{2},\;\left\{a\right\}=\left\{10,13,17,22,28,37\right\},$$

where *S*_Original_ is the actual area of all six spheres together. The scan resolution can be acquired from *Amide* software where each pixel size is 4.6875 × 4.6875 mm and each slice size is 128 × 128 pixels. The overall slice area and the ratio between both areas can be calculated according to (16), respectively, where SBR is the spheres to background ratio

(16) $$\begin{array}{cc} A_{\text{Original}} & =128\times 128\times 4.6875\times 4.6875=360000\;{\text{mm}}^{2},\\ \text{SBR} & =\frac{S_{\text{Original}}}{A_{\text{Original}}-S_{\text{Original}}}\times 100\%=0.702\%. \end{array}$$

**Figure 18:**
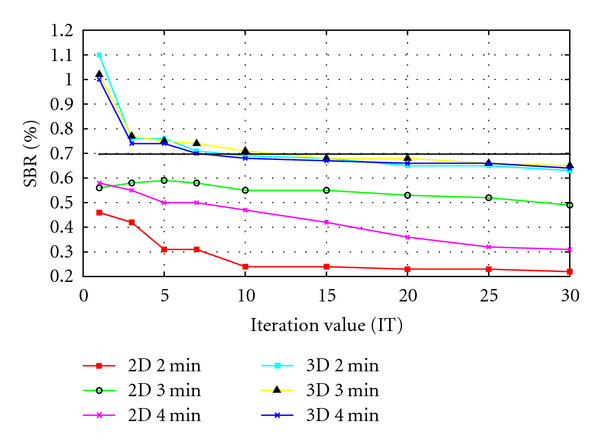
Scanner variables effects on the segmented image.

Table 4 illustrates the results for a sample data provided from the collaborator for different scanner variables. It can be seen that the SBR percentages varies based on the scanner variables used. To explain the effects of those variables on the output image, Figure 18 illustrates the changes in the quality of the segmented image based on the scanners variables.

**Figure 19:**
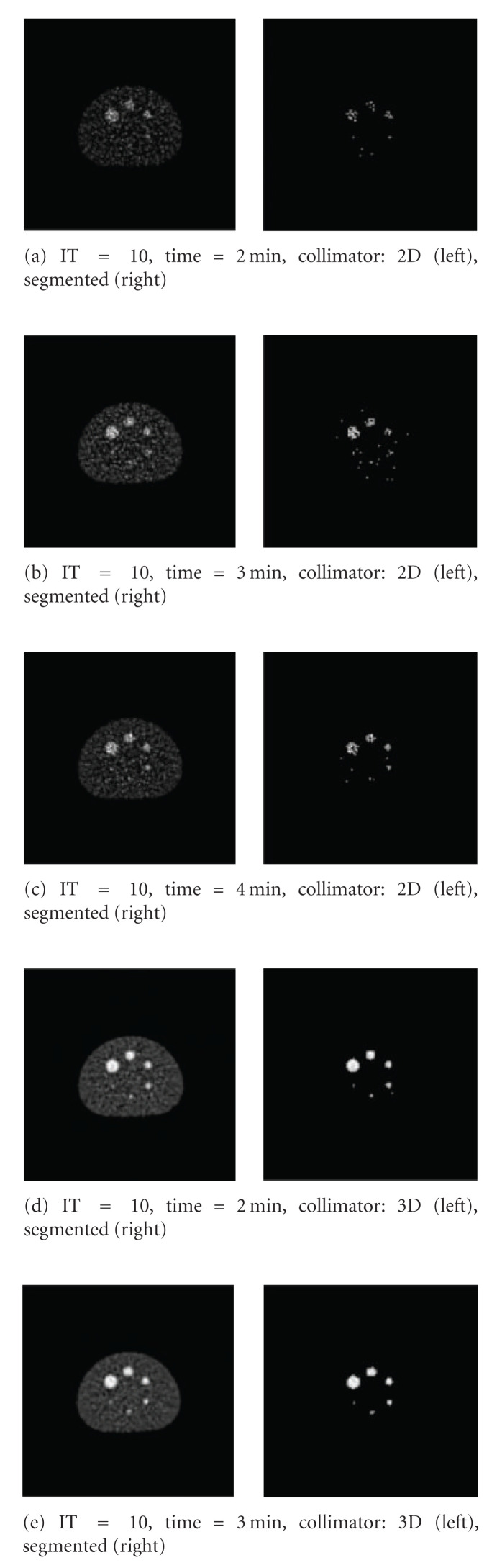

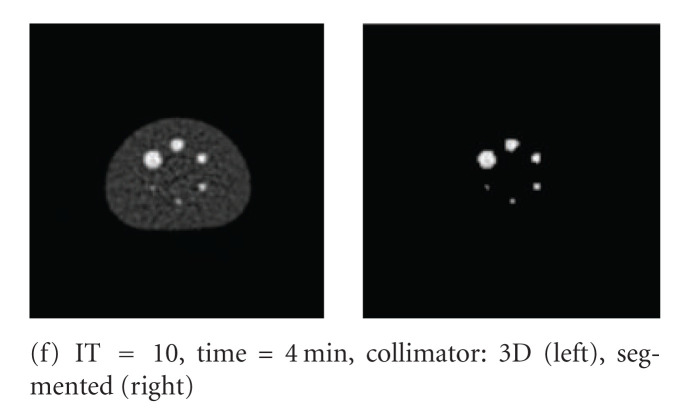
Segmented results achieved at IT value (10), where the best results detected.

It can be seen from Figure 18 that 3D scans produce closer SBR percentages than 2D for all iterations except at IT = 1. It can be noticed that the area evaluating the spheres decreases as the IT value increases for both 2D and 3D and for all bed section scanning times. These results match the expectation of the radiologists at Paul Strickland Scanner Centre. The segmented results achieved at IT = 10 are illustrated in Figure 19.

**Figure 20:**
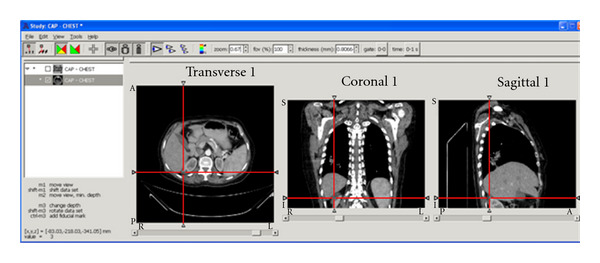
AMIDE snapshot locating the kidney cancer.

**Figure 21:**
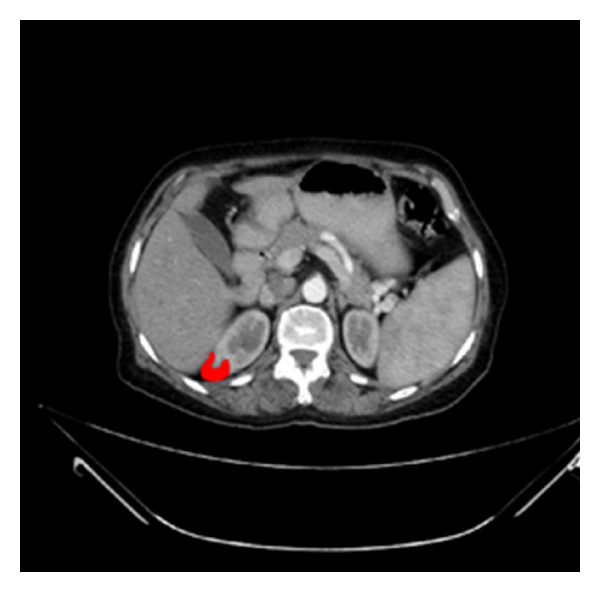
ROI highlighted in the original image (kidney cancer).

A predefined clinical dataset comprised of 217 slices, with slice thickness of 3.0 mm has been tested on the proposed system. Based on the provided report, the patient is affected by multiple bilateral renal cortical cysts; the largest one is seen in the lower pole of the right kidney, measuring about 47 × 45 mm. A snapshot taken for a DICOM viewer window and the ROI has been located by the red lines in three different orientations of the patient's body scan (Figure 20). It can be seen that ROI appears more clearly in its biggest illustration in slice 198; this slice is illustrated in Figure 21, and the ROI (kidney cancer) has been highlighted by red colour.

**Figure 22:**
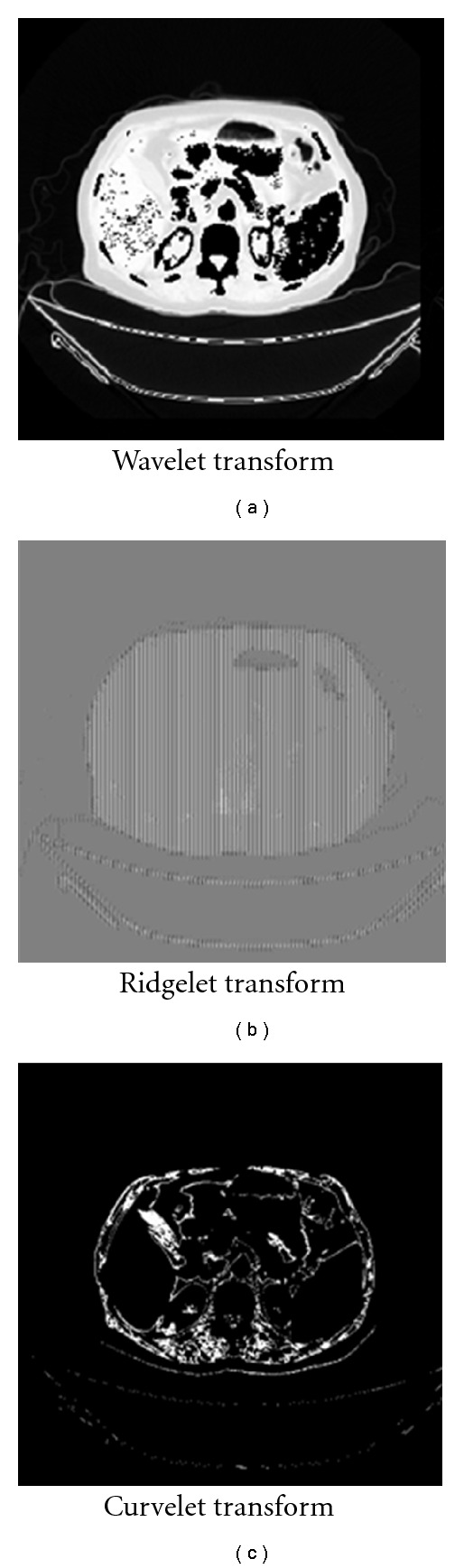
MRA for real clinical data segmentation.

MRA have been applied on the medical image to segment it and detect ROI.  Figure 22 illustrates the outputs of applying those techniques.

The performance of the proposed techniques for segmenting the illustrated slice in Figure 21 is explained in Table 5. The area of the cancer has been measured and compared to the provided report and then used to qualify the performance of each technique as well as MSE, data loss, and PSNR. 

**Figure 23:**
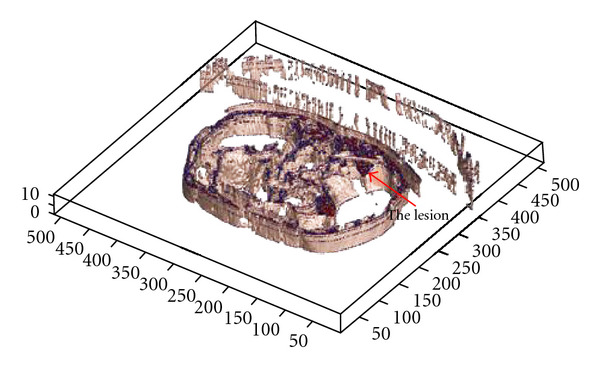
Segmenting patient volume data affected by the kidney cancer.

**Figure 24:**
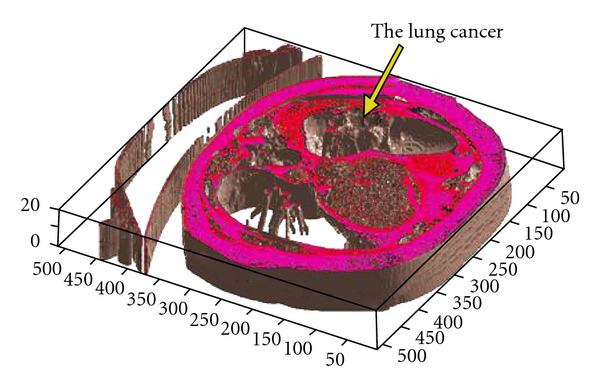
Segmenting patient volume data affected by the lung cancer.

**Figure 25:**
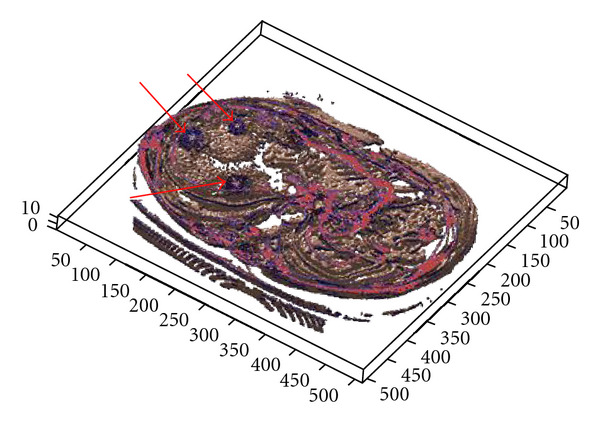
Segmenting patient volume data affected by the liver cancer (located by red arrows).

The clinical datasets have been segmented also using 3D segmentation techniques, and the lesions were detected accurately. Curvelet transform has been used before 3D segmentation to achieve a denoised CT output and ensure smoother edges. Patient data which includes lesions in liver, kidney and lung has been segmented and visualized in Figures 23, 24, and 25, where the OOIs are located.

## 5. Conclusion

Due to the changing shapes of organs in medical images, segmentation process using multiresolution analysis combined with thresholding as pre- and postprocessing step allows accurate detection of ROIs. Multiresolution analysis such as wavelet transform is extensively used in medical image segmentation and provides better accuracy in results. Curvelet and ridgelet transforms are new extension of the wavelet transform that aims to deal with interesting phenomena occurring along higher dimensional singularities. Though wavelets are well suited to point singularities, they have limitations with orientation selectivity hence do not represent changing geometric features along edges effectively. Curvelet transform exhibits good reconstruction of the edge data by incorporating a directional component to the traditional wavelet transform. Experimental study in this report has shown that curvelet-based segmentation of the medical images not only provide good-quality reconstruction of detected ROI, promising results are also achieved in terms of accurately detecting ROI and denoising process. Curvelet transform is a new tool and utilization of this technique; it is far from sufficient in the medical image processing area. The future work related to this is the implementation of 3D MRA transform which can be applied directly on medical volumes to detect obstacle and objects of interest. 

## Figures and Tables

**Table 1:** Wavelet and ridgelet comparisons depending on SNR and processing time.

Domain	Wavelet			Ridgelet			Spatial
	Level 1	Level 2	Level 3	*P* = 5	*P* = 11	*P* = 31	
SNR (dB)	10.63	11.14	10.95	10.37	11.43	11.88	7.17
Time (sec)	0.23	0.24	0.50	71.5	29.91	10.01	1.18

**Table 2:** The error percentages of spheres diameters measurements for NEMA IEC body phantom.

Spheres (mm)	Error % for measured diameters					
	10	13	17	22	28	37
K-means [3]	−13.6	−11.5	−5.77	−5.51	−5.1	−5.01
MRFM [3]	−7.41	−8.69	4.28	4.06	3.9	3.89
Clustering [3]	18.6	16.0	9.0	7.5	5.5	1.1
Iterative Thresholding [3]	3.0	3.1	0.6	0.9	1.1	1.8
Wavelet						
Haar						
Level 1	−2.9	−2.46	1.35	0.82	0.29	0.05
Level 2	−10.9	−6.67	3.88	−1.3	−0.76	−1.95
Level 3	—	—	5.65	−18.2	2.57	−3.24
Daubechies						
Level 1	−7.43	−2.69	0.12	2.0	2.17	1.81
Level 2	−5.2	0.15	−4.24	0.73	0.62	−0.11
Ridgelet	−10.93	−6.67	3.88	−1.30	−0.76	−1.95
Curvelet	2.65	1.62	1.07	−0.82	−0.33	−0.09

**Table 3:** Comparison of curvelet, ridgelet, and wavelet denoising in terms of PSNR and MSE.

Image name	Curvelet denoising		Ridgelet denoising		Wavelet denoising	
	MSE	PSNR (dB)	MSE	PSNR (dB)	MSE	PSNR (dB)
NEMA	41.67	31.93	108.78	26.14	101.12	28.08
Chest	58.8	30.44	152.45	23.55	147.63	26.44

**Table 4:** Spheres to background ratio (SBR) for different variable samples.

2D/3D	Time/bed section	Iteration	SUB	SBR (%)
2D	2 min	1	30	0.46
2D	2 min	5	30	0.31
2D	2 min	10	30	0.24
2D	2 min	30	30	0.22
2D	3 min	1	30	0.56
2D	3 min	5	30	0.59
2D	3 min	10	30	0.55
2D	3 min	20	30	0.53
2D	4 min	3	30	0.55
2D	4 min	15	30	0.42
2D	4 min	20	30	0.36
2D	4 min	30	30	0.31
3D	2 min	1	32	1.1
3D	2 min	3	32	0.76
3D	2 min	7	32	0.71
3D	2 min	10	32	0.69
3D	3 min	1	32	1.02
3D	3 min	3	32	0.77
3D	3 min	10	32	0.71
3D	3 min	15	32	0.68
3D	4 min	3	32	0.74
3D	4 min	10	32	0.68
3D	4 min	30	32	0.64

**Table 5:** Segmentation techniques' performance based on patient data (kidney cancer data).

Segmentation technique	Cancer area accuracy (%)	MSE	PSNR (dB)	Data loss
DWT				
Haar	91.0	102.7	35.2	Normal
Daubechies	89.5	104.5	34.3	Normal
Wavelet Packet	83.2	111.2	30.9	Normal
Ridgelet	—	109.9	30.3	High
Curvelet	96.2	88.2	29.5	Normal
